# Dabigatran-Related Nephropathy in a Patient with Undiagnosed IgA Nephropathy

**DOI:** 10.1155/2015/298261

**Published:** 2015-08-05

**Authors:** Rachele Escoli, Paulo Santos, Sequeira Andrade, Fernanda Carvalho

**Affiliations:** ^1^Department of Nephrology, Centro Hospitalar do Médio Tejo, 2350-754 Torres Novas, Portugal; ^2^Department of Nephrology, Centro Hospitalar de Lisboa Central, Hospital Curry Cabral, 1069-166 Lisbon, Portugal

## Abstract

Dabigatran is a direct thrombin inhibitor used as an alternative to warfarin for long term anticoagulation. Warfarin-related nephropathy is an increasingly recognized entity, but recent evidence suggests that dabigatran can cause a WRN-like syndrome. We describe a case of a biopsy-proven anticoagulant nephropathy related to dabigatran in a patient with IgA nephropathy and propose that, despite the base glomerular disease, acute kidney injury was due to tubular obstruction by red blood cells and heme-associated tubular injury, and through a mechanism involving inhibition of anticoagulation cascade and barrier abnormalities caused by molecular mechanisms.

## 1. Introduction

Anticoagulant therapy plays a central role in the prevention and treatment of venous and arterial thromboembolic diseases. Recently, several oral anticoagulants were approved, including the direct thrombin inhibitors such as dabigatran. It has a quick onset of action, results in a predictable anticoagulation response, and does not require routine laboratory monitoring. However, concerns have been raised since there is no antidote for treatment of secondary hemorrhages. We report a case of a 69-year-old woman with a biopsy-proven anticoagulant nephropathy related to dabigatran and discuss the diagnostic and management approach.

## 2. Case Presentation

A 69-year-old white female with a past history of hypertension presented with nausea, vomiting, and oliguria. The patient had been in her usual state of health until 2 weeks earlier, when she developed palpitations that prompted her to seek medical care. New-onset atrial fibrillation was diagnosed. After reversing into sinus rhythm with amiodarone, she was discharged with a prescription of dabigatran 110 mg twice daily (Pradaxa Boehringer). At this time serum creatinine was 1,5 mg/dL (corresponding to an estimated glomerular filtration rate [eGFR] of 35,2 mL/min/1,73 m^2^ as calculated by the CKD-EPI [Chronic Kidney Disease Epidemiology Collaboration] equation). Two weeks later she started vomiting and having oliguria and was sent to our medical facilities. She denied additional complaints and was on dabigatran 100 mg twice a day during the previous two weeks.

The patient's medical history included arterial hypertension medicated with ramipril. On admission blood pressure was 212/98 mmHg, pulse rate was 98 heart beats per minute, and she was oliguric. The physical examination revealed hydrated mucosa with no respiratory distress, crackles in bilateral lung fields, and mild lower-extremity edema. Laboratory results showed the following: serum urea was 230 mg/dL, serum creatinine was 8 mg/dL, hemoglobin was 9.1 g/dL, white blood cell count was 14.7 × 10^3^/*μ*L with 86% of PMN and 0.2% of eosinophils, platelet count was 369 × 10^3^/*μ*L, prothrombin time (PT) was 25.3 seconds with international normalized ratio (INR) of 2.3, activated partial thromboplastin time (aPTT) was 68 seconds, LDH was 531 IU/L, and C-reactive protein was 7.1 mg/dL. A random urine specimen revealed that leukocytes were 500/*μ*L, proteins were 100 mg/dL, and hemoglobin was 1 mg/dL. Urine sediment had hematuria (>100 red blood cells (RBC)/high-power field) and leukocyturia (6 leukocytes/high-power field) without dysmorphic red blood cells or red blood casts. Renal ultrasound suggested globular kidneys with regular shape but hyperechogenic cortical parenchyma without hydronephrosis. The patient was then transferred to the Nephrology Department. Due to oliguric acute renal failure she started hemodialysis two days after being admitted and proceeding with investigation. A peripheral blood smear did not show schizocytes. C3 complement level was decreased (52 mg/dL). C4 and antistreptolysin were normal. Screening for antinuclear antibody, antineutrophil cytoplasmic antibody, anti-glomerular basement membrane antibody, cryoglobulins, and antiphospholipid antibody and VDRL test were negative. After an inconclusive Doppler renal ultrasound, a contrast enhanced computed tomography angiography was performed four days after admission and showed no abnormalities. A transthoracic echocardiogram was done and illustrated normal sized left chambers without intracardiac thrombus. After 3 sessions of hemodialysis a normal aPTT was accomplished and due to persistent oliguria a biopsy was performed.

**Figure 1:**
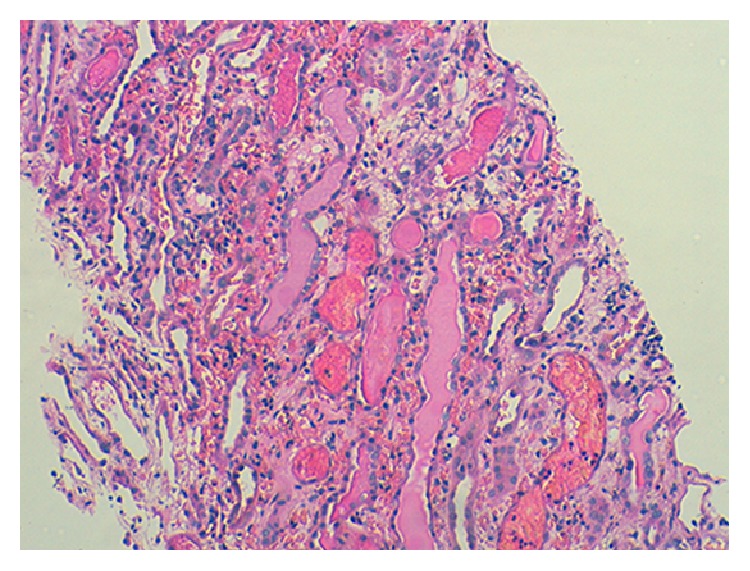
Prominent interstitial hemorrhage and intratubular casts (haematoxylin/eosin staining, magnification 100x).

**Figure 2:**
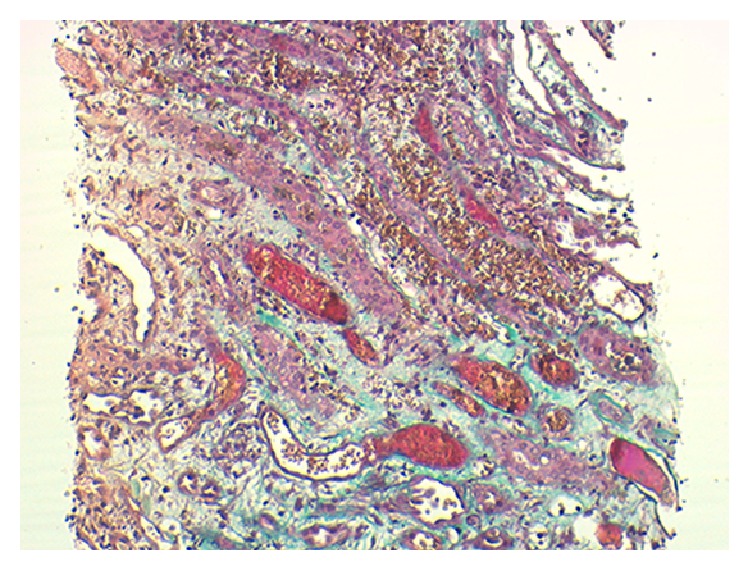
Interstitial hemorrhage (Masson's trichrome, magnification 100x).

**Figure 3:**
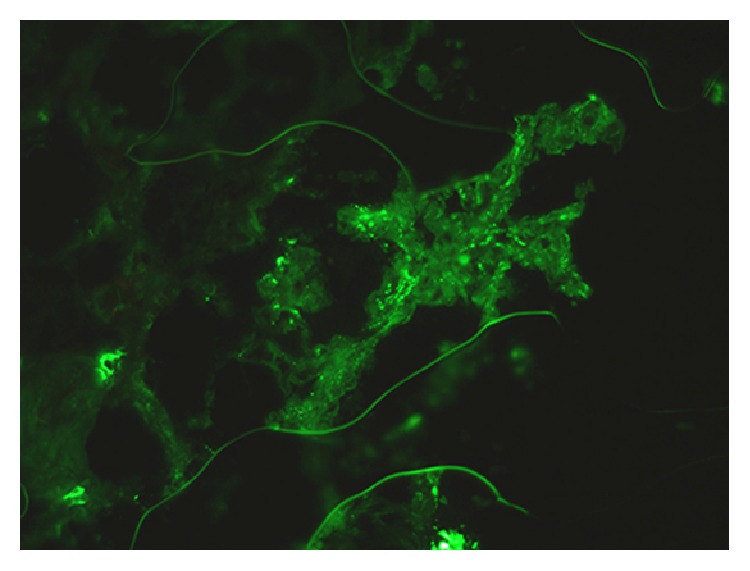
Direct immunofluorescence showing granular mesangial staining for IgA in the expanded mesangium of the biopsy, magnification 400x.

Five glomeruli appeared normal. The tubulointerstitium had large intratubular RBC casts, extensive tubular necrosis, and interstitial hemorrhage (Figures 1 and 2). By immunofluorescence there were mesangial deposits of IgA (++), C3 (++), K, and *λ* chains on 3 glomeruli (Figure 3).

So the diagnosis of IgA nephropathy, anticoagulant nephropathy with acute tubular necrosis, and interstitial hemorrhage was made. Following the kidney biopsy there were perirenal haematoma and hypotension. Three units of RBC were provided and resolution was achieved under tight follow-up. After intravenous fluid reposition she restored diuresis (hematuria). Two weeks later, renal function improved, urine cleared, and patient was discharged. Creatinine was 1.9 mg/dL in the last clinical evaluation.

## 3. Discussion

Anticoagulant-related nephropathy (ARN) is a form of acute kidney injury caused by excessive anticoagulation first described with warfarin, and because of that it is called warfarin-related nephropathy (WRN) [1]. Diagnosis should be suspected among patients who present with unexplained acute renal injury defined as a serum creatinine increase greater than 0.3 mg/dL within one week of an INR measurement greater than 3 in a patient treated with warfarin, excluding other causes of AKI and bleeding [1, 2]. Recent evidence suggests that WRN-like syndromes are not confined to anticoagulation with warfarin but may occur with other anticoagulants, such as acenocoumarol [3] and dabigatran [2].

In WRN AKI occurs through glomerular hematuria with subsequent widespread tubular obstruction [4]. Biopsy studies showed RBCs in tubules and occlusive RBCs casts predominantly in distal nephron segments [4, 5]. Several pathogenic mechanisms were proposed. The combination of even mild glomerular disease and warfarin-induced coagulopathy seems to be the key point [4]. This leads to glomerular hematuria and to a significant accumulation of RBCs within nephrons that form occlusive casts, especially when urinary flow is diminished [4, 6]. Although glomerular hematuria is essential, it seems that interstitial hemorrhage may also have an important role [3]. So the dominant mechanism of AKI in WRN is probably tubular obstruction by RBC casts, which, associated with interstitial hemorrhage, leads to increased oxidative stress in the kidney [7, 8].

There are many underlying risk factors for WRN, such as age, CKD, due to higher risk of supratherapeutic INR, diabetes and diabetic nephropathy, hypertension, and heart failure [5].

Dabigatran is an anticoagulant used for stroke prevention in atrial fibrillation [9]. Recent evidence suggests that dabigatran has many hemorrhagic complications. However, in what concerns kidney involvement, information is scarce [10]. Dabigatran has 80% renal elimination and is not recommended for patients with creatinine clearance less than 15 mL/min or on dialysis, needing a dose adjustment in patients with creatinine clearance between 15 and 30 mL/min, in order to reduce hemorrhagic complications [11].

Evidence from animal studies revealed that dabigatran may cause AKI by two major pathogenic mechanisms: first, tubular obstruction by RBCs and, second, a mechanism possibly involving protease-activated receptor 1 (PAR-1) [1]. PAR-1 is a G protein-coupled receptor that participates in the regulation of the endothelial functions, vascular permeability, leukocyte migration, and adhesion and is the major effector of thrombin signaling [3]. Either vitamin K antagonists or direct thrombin inhibitors decrease thrombin activity. By acting on thrombomodulin, thrombin activates protein C and modulates the anticoagulation cascade. The same happens with PAR-1. In the aforementioned study the authors proposed that thrombin plays an important role in the glomerular filtration barrier function, and its decreased activity (secondary to anticoagulation) results in glomerular filtration barrier abnormalities. Indeed, treatment with selective PAR-1 inhibitor results in increased creatinine, hematuria, and tubular RBC casts, findings similar to those in animals with WRN or treated with dabigatran. These effects are similar to WRN. However in contrast to WRN, where kidney injury was seen only in animals with CKD, the effects of dabigatran were prominent in control rats as well. These findings suggest that the kidney risk with dabigatran may be greater than that of warfarin [3].

To the best of our knowledge, only two cases of dabigatran-induced AKI have been reported [12, 13]. In both cases patients presented with hematuria and had histologic evidence of hemorrhage into renal tubules. In the Moeckel et al. clinical report the patient had previously mild undiagnosed IgA nephropathy [13], as presented in our case. This raises the question if IgA nephropathy is a risk factor or a predisposing condition in anticoagulated patients with dabigatran as was described in WRN. The main clinical feature of IgA nephropathy is hematuria, which can be micro- or macroscopic, both unnoticed by our patient, and it is plausible to think that an entity, which, by nature, already predisposes hematuria, may be related or may be a risk factor to ARN. Fundamental WRN pathological lesions as described above were also observed in our patient, which suggests that the physiopathological mechanisms that induce AKI may have a common way, particularly with regard to tubular obstruction and interstitial hemorrhage. In our patient's case we propose that the combination of IgA nephropathy which associated with the age, CKD, and the medical history of hypertension possibly leads to the perfect background.

Concerning treatment, restoring the aPTT into a therapeutic range while doing supportive renal treatment is primordial [1]. In what concerns dabigatran-related AKI hemodialysis seems to be effective [9, 13, 14]. About the prognosis, there seem to be discrepancies between WRN and dabigatran-related AKI. The clinical outcome in a WRN study was unfavorable: 66% of patients did not recover baseline function [4]. With regard to the two reported cases of dabigatran-induced AKI, in both renal function improved [12, 13]. In our case, there seems to be a recovery of renal function and we think that the absence of histological markers of poor prognosis of IgA nephropathy, such as the lack of interstitial fibrosis, tubular atrophy, glomerular sclerosis, or endocapillary hypercellularity, may possibly have contributed to this good outcome. The short period of administration and the lower dose of dabigatran may also have had influence. However, since there are so few published cases of dabigatran-related nephropathy, it is impossible to compare outcomes of these two entities.

This raises the note of caution about oral anticoagulation in patients with kidney disease. They are at a higher risk of overanticoagulation, gross hematuria, and AKI [15]. So physicians involved in the clinical management of anticoagulated patients should be aware of the ARN, either by warfarin or other anticoagulants like dabigatran. A correct coagulation and kidney function monitoring is required and, if needed, anticoagulants should be stopped or decreased [15].
